# Analysis of Volatile Compounds during Food Fermentation

**DOI:** 10.3390/foods12193635

**Published:** 2023-09-30

**Authors:** Laura Vázquez-Araújo

**Affiliations:** 1 BCC Innovation, Technology Center in Gastronomy, Basque Culinary Center, 20009 Donostia-San Sebastián, Spain; lvazquez@bculinary.com; 2 Basque Culinary Center, Faculty of Gastronomic Sciences, Mondragon Unibertsitatea, 20009 Donostia-San Sebastián, Spain

Fermented foods from different raw materials (fruits, cereals, milk, etc.) have been consumed by humanity for over 10,000 years [1]. Still, research carried out in this product category has increased substantially in recent years, multiplying by 16 the number of scientific publications in Scopus from 2003 to 2023. It has been reported that the discovery of fermentation was an accident when humans attempted to preserve foods, and the result was a product significantly transformed by microorganisms. Still, since the 19th century, with the understanding of Pasteurization, controlled fermentations have been carried out to develop specific products with specific sensory properties [2]. Different areas of the world are characterized by using different substrates, using microorganisms differently, and having other fermentation-related practices. However, some common organoleptic and culinary aspects can be identified in different countries and cultures [3]. Both in spontaneous fermentations and those driven by specific microorganisms, the transformation process of the raw material involves a significant modification of its texture and aromatic profile, resulting in what can be considered a new food or beverage. The sensory shift of fermented foods, as well as their increased shelf life and potential health benefits, are key factors that have attracted the attention of consumers, chefs, and the food industry around the world in recent years.

Different groups of volatile compounds have been studied as responsible for specific sensory properties of fermented foods. Alcohols and esters, some of the main contributors to the flavor of beer, sake, and wine, are produced by yeasts during the fermentation process and give the beverages their characteristic fruity, candy, and perfume-like aromas [4]. Volatile sulfur compounds, including thiols, sulfides, thioethers, and esters, have been reported to have a significant role in the flavor of cheese and fermented beverages such as beer or wine [5]. Various strains of lactic acid bacteria have been shown to impact in different manners the volatile profile of fruits and vegetables, modifying the concentration of aldehydes, alcohols, ketones, and terpenoids [6]. Although some research in this matter has been conducted, much is still unknown on the volatile transformation of different matrices and how to use fermentation to design new flavors adapted to consumers’ desires.

Traditional fermentation processes, such as those conducted by households, small producers, and restaurants, and also precision fermentation processes developed by industry, could be improved by gaining knowledge on how to control and modulate the generation of specific volatile compounds. The utility of bioinformatics and advanced statistical and artificial intelligence tools have also been suggested as innovative approaches to better understand and predict how different microorganisms will transform raw materials into new aromas [7]. The role of different microbial communities and species in generating or modulating the presence of specific volatile compounds in fermented foods can now be studied using molecular and omics approaches. In addition to physico-chemical and analytical tools used to determine the volatile dynamics and profile of foods, sensory science methods are also needed to identify and understand how human beings perceive these compounds. This is essential to get a complete overview of the aroma of fermented foods.

Because of all these reasons, the present Special Issue collects a set a set of manuscripts that present data on (1) the volatile composition of fermented foods, either made using traditional or advanced biotechnological methods; (2) different methods to correctly identify and quantify volatile compounds during fermentation processes; (3) the relationships among volatile compounds and perceived aromas in fermented products; and (4) other aspects of food fermentation that involve its volatile composition. Therefore, this Special Issue will be of interest to food quality control professionals, food designers, sensory and consumer scientists, and fermented food producers (such as brewers and chefs) who are interested in learning more about how to control and improve the aroma of their products.

## References

[1] Liu L., Wang J., Rosenberg D., Zhao H., Lengyel G., Nadel D. (2018). Fermented beverage and food storage in 13,000 y-old stone mortars at Raqefet Cave, Israel: Investigating Natufian ritual feasting. J. Archaeol. Sci. Rep..

[2] Ross R.P., Morgan S., Hill C. (2002). Preservation and fermentation: Past, present and future. Int. J. Food Microbiol..

[3] Tamang J.P., Cotter P.D., Endo A., Han N.S., Kort R., Liu S.Q., Mayo B., Westerik N., Hutkins R. (2020). Fermented foods in a global age: East meets West. Compr. Rev. Food Sci. Food Saf..

[4] Saerens S.M., Delvaux F.R., Verstrepen K.J., Thevelein J.M. (2010). Production and biological function of volatile esters in *Saccharomyces cerevisiae*. Microb. Biotechnol..

[5] Landaud S., Helinck S., Bonnarme P. (2008). Formation of volatile sulfur compounds and metabolism of methionine and other sulfur compounds in fermented food. Appl. Microbiol. Biotechnol..

[6] Lorn D., Ho P.H., Tan R., Licandro H., Waché Y. (2021). Screening of lactic acid bacteria for their potential use as aromatic starters in fermented vegetables. Int. J. Food Microbiol..

[7] Galimberti A., Bruno A., Agostinetto G., Casiraghi M., Guzzetti L., Labra M. (2021). Fermented food products in the era of globalization: Tradition meets biotechnology innovations. Curr. Opin. Biotechnol..

